# Treg-driven tumour control by PI3Kδ inhibition limits myeloid-derived suppressor cell expansion

**DOI:** 10.1038/s41416-022-01917-0

**Published:** 2022-08-19

**Authors:** Sarah N. Lauder, Kathryn Smart, Valentina M. T. Bart, Ana Pires, Jake Scott, Stefan Milutinovic, Andrew Godkin, Bart Vanhaesebroeck, Awen Gallimore

**Affiliations:** 1 Division of Infection and Immunity, Cardiff University School of Medicine, SIURI, Cardiff, CF14 4XN UK; 2 UCL Cancer Institute, Paul O’Gorman Building, University College London, 72 Huntley Street, London, WC1E 6BT UK

**Keywords:** Tumour immunology, Tumour immunology

## Abstract

**Background:**

Recent studies have demonstrated that blocking the PI3Kδ signalling enzyme (by administering a small molecule inhibitor, PI-3065) can potently improve the anti-tumour T-cell response through direct inhibition of Tregs. This treatment also has a negative impact on MDSC numbers but the primary mechanism driving this effect has remained unclear.

**Methods:**

The 4T1 breast cancer mouse model was used in combination with PI-3065 to gain insights into the effect of PI3Kδ inhibition on MDSCs.

**Results:**

PI-3065 treatment resulted in a concomitant reduction in MDSC expansion and tumour size. However, targeting Tregs independent of PI-3065 was also associated with reduced tumour volume and MDSC numbers. Surgical removal of tumours resulted in a rapid and significant decline in MDSC numbers, whilst ex vivo studies using cells from PI-3065-treated mice demonstrated no direct effect of the inhibitor on MDSC activity.

**Conclusions:**

Our data suggest that MDSCs are not inhibited directly by PI-3065 treatment but that their reduced recruitment and immunosuppression within the tumour microenvironment is an indirect consequence of PI3Kδ-inhibition-driven tumour control. This indicates that PI3Kδ inhibition drives tumour immunity by breaking down multiple immunosuppressive pathways through both direct mechanisms (on Treg) and indirect mechanisms, secondary to tumour control (on MDSCs).

## Introduction

Immunotherapy can result in the eradication of tumour burden, however, for the majority of patients a sufficient T-cell response fails to develop and ultimately treatment is unsuccessful. Treatment failure is partly driven by immunosuppressive cells such as myeloid-derived suppressor cells (MDSCs) and regulatory T cells (Tregs) which restrain the activities of anti-tumour CD8^+^ T cells. Elevated numbers of both Tregs and MDSCs occur in the peripheral blood of cancer patients, including breast cancer patients, and have been associated with cancer stage and poor prognosis [1–5]. Thus, successful targeting of MDSCs and Tregs through depletion or inhibition is an important goal for cancer therapy.

MDSCs are a population of cells of myeloid origin with potent immunosuppressive capacity, particularly in the tumour microenvironment [6]. In mice, there are two main subpopulations of MDSCs, all of which are CD11b^+^, namely monocytic-MDSCs (M-MDSCs), characterised by the expression of the phenotypic markers Ly6G^−^ Ly6C^hi^, and polymorphonuclear-MDSCs (PMN-MDSCs), delineated by Ly6G^+^ Ly6C^lo^ expression [7, 8]. MDSCs express high levels of Arginase-1 (Arg-1), Nitric Oxide (NO) reactive oxygen species (ROS), and secrete IL-10 and TGFβ, all of which play a role in suppressing T-cell function [9]. Furthermore, studies to date have demonstrated that MDSCs promote T-cell immunosuppression through their ability to induce and recruit suppressive Tregs [10–12]. Whilst MDSCs appear to induce *de novo* Treg expansion, recent evidence points to reciprocity between MDSCs and Tregs, whereby Treg depletion results in reduced numbers of both PMN-MDSCs and M-MDSCs, suggesting that Tregs support the survival and expansion of MDSCs [13].

Several studies have demonstrated that targeting phosphoinositide 3-kinase δ (PI3Kδ) can improve anti-tumour immune responses [14]. Tregs are reliant on PI3Kδ and genetic or pharmacological inhibition of PI3Kδ significantly reduce Treg proliferation and recruitment [15–19]. MDSCs use PI3Kδ alongside the PI3Kγ isoform for survival and function [20]. Consequently, targeting both Tregs and MDSCs by inhibiting PI3Kδ signalling has the potential to unleash powerful anti-tumour T-cell responses and ultimately control tumour burden.

We and others recently demonstrated that the PI3Kδ-selective inhibitor PI-3065, reduces the expansion and suppressive capacity of Tregs, conferring CD8^+^ T-cell-mediated tumour control in the 4T1 mouse model of breast cancer [17, 18]. As it is known that 4T1 tumours are associated with a significant expansion of MDSCs in vivo, we now used this model to examine how treatment with PI-3065 alters MDSC behaviour. We specifically sought to determine whether pharmacological inhibition of PI3Kδ affects MDSCs directly or indirectly as a result of alleviating the tumour-promoting effects of Tregs.

## Materials and methods

### Tumour model

Adult (8-week-old) female BALB/c and BALB/c Nude (CAnN.Cg-Foxn1/Crl) mice were purchased from Charles River, Depletion of REGulatory T-cell (DEREG) mice, a strain of BALB/c mice developed using a Bacterial Artificial Chromosome (BAC) containing the *foxp3* locus and a diphtheria toxin receptor (DTR)-eGFP fusion protein inserted into the first exon of the *foxp3* gene [21], were bred and housed in filter-top cages in specific pathogen-free conditions, with standard chow and water provided ad libitum. Experiments were conducted in accordance with Home Office UK guidelines. Mice were randomly assigned to treatment groups by animal technicians who were not directly involved in the study, researchers were not blinded to treatment groups. The 4T1 breast cancer cell line was obtained from ATCC (CRL-2539) and maintained in culture medium (RPMI 1640, 10% FCS, 2 mM l-glutamine, 1 mM sodium pyruvate, and 50 mg/ml penicillin-streptomycin). In all, 1 × 10^5^ 4T1 cells were injected subcutaneously into the mammary fat pad. Tumours were measured using digital callipers from day 7 up to three times per week until the mouse was sacrificed. The following calculation was used to determine tumour volume: (length ×  width × short)*(3.14/6), [where short equals the lower of the length and width measurements and provides an estimate of height]. Tumour and spleen weight was measured at endpoint following excision of the tumour from the host.

### In vivo drug treatment

PI-3065 was provided by Genentech and administered by oral gavage at a dose of 75 mg/kg, with vehicle-treated mice given an equivalent volume of carrier solution, as described previously [17]. Mice were dosed daily from day −1 prior to tumour injection until the termination of the experiment. As described previously, the Tregs of DEREG mice were selectively ablated by injection of diphtheria toxin (DT) three times per week from day 5 post tumour injection [21].

### Tissue dissociation

Spleens were isolated from tumour-bearing mice, mechanically disaggregated and then passed through a 70-μm filter, prior to lysis of red blood cells using RBC lysis buffer (Biolegend).

### Flow cytometry

Cells were washed twice with PBS and stained using LIVE/DEAD Aqua (Invitrogen) according to the manufacturer’s instructions. Cells were washed twice with FACS buffer and Fc receptors blocked with anti-CD16/32 (clone 93; Biolegend). Cells were surface stained with the following antibodies (all from Biolegend): CD11b (M1/70, PE), CD11c (N418, BV605), Ly6G (1A8, FITC, BV421), Ly6C (HK1.4, APC), CD45 (30-F11, APC/Fire 750, PE-Cy7), c-KIT (ACK2, BV421), NK1.1 (PK136, APC/Fire 750), CD19 (ID3/CD19, PE), CD3 (17A2, BV785), CD8 (53–6.7, BV421), CD4 (GK1.5, FITC), FoxP3 (FJK-16s, APC).

### Tumour resection surgery

Tumours of comparable sizes were removed at day 18 post tumour injection. Mice were anaesthetised (isofluorane) and given a subcutaneous injection with the anti-inflammatory analgesic, Metacam (Boehringer Ingelheim). The tumour and the surrounding area were shaved and swabbed with Hibiscrub antibacterial wash and Surgical Spirit. Aseptic techniques were used to excise the tumour and close the wound using horizontal mattress sutures (Ethicon). Mice, placed in a heated recovery chamber until consciousness was regained, were monitored postoperative for well-being and suture strength. At day 21 (3 days post resection) and day 28 (10 days post resection), spleens were isolated from both resected mice and tumour-bearing mice at the equivalent time points and the splenic MDSC populations enumerated.

### T-cell/MDSC co-culture proliferation assay

CD8^+^ T cells were isolated from naive spleens using a negative magnetic bead selection kit (Biolegend). MDSCs were isolated from the spleens of mice bearing 4T1 tumours that had either been treated with vehicle or PI-3065 using a negative magnetic bead selection kit (Stemcell). CD8^+^ T cells were labelled with Tag-It-Violet (Biolegend) prior to stimulation with plate-bound anti-CD3 10 μg/ml (Biolegend, 145–2C11), 1 μg/ml soluble anti-CD28 (Biolegend, 37.51) and 30 IU/ml IL-2. MDSCs were added to the CD8^+^ T cells at a ratio of either 1:1 (MDSCs:CD8) or 4:1. Cocultures were left for 3 days prior to flow cytometric analysis.

### Statistical analyses

**Fig. 1:**
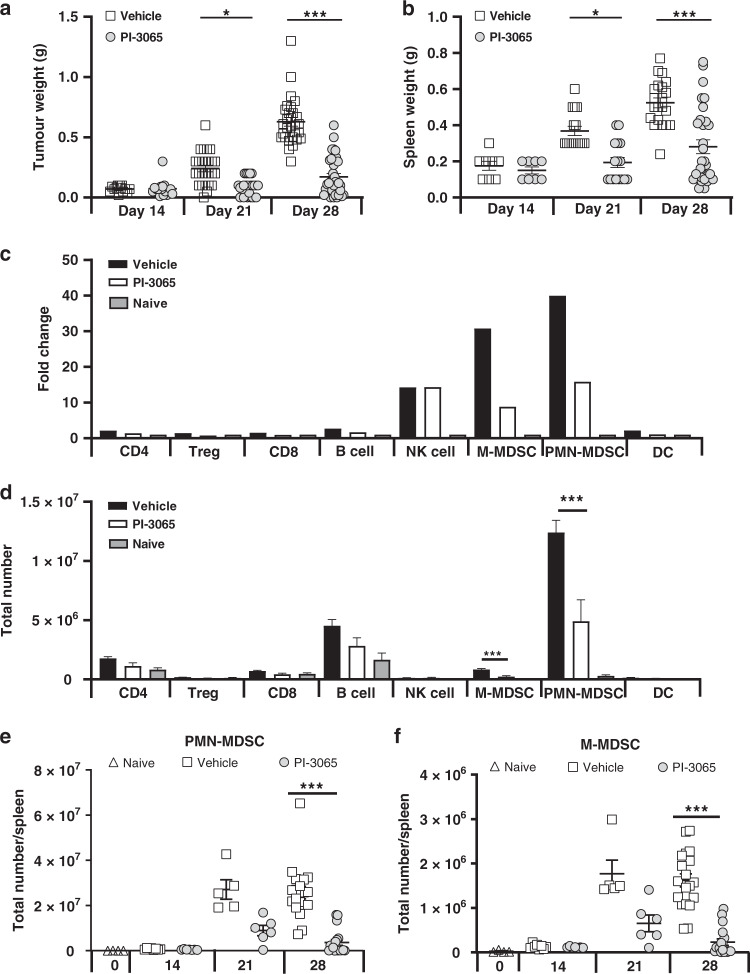
PI3Kδ inhibition controls tumour-driven splenomegaly. **a** Tumour burden and **b** spleen weight at day 14, 21 and 28 post tumour injection in mice treated with either vehicle or PI-3065 (13–36 mice/group/timepoint). **c** Fold change compared to tumour-naive mice of spleen immune cell populations in vehicle or PI-3065-treated mice at day 28 post tumour injection (*n* = 8 mice/group). **d** Total number of immune cell populations in the spleen of tumour-naive or vehicle and PI-3065-treated mice at day 28 post tumour injection (*n* = 8 mice/group). Total numbers of splenic (**e**) PMN-MDSC and (**f**) M-MDSC at days 14, 21 and 28 post tumour injection in mice treated with either vehicle or PI-3065 (5–19 mice/group/timepoint). All data are displayed as the mean ± SEM. Statistical significance was determined by Kruskal–Wallis test (**a**, **b**) and one-way ANOVA (**e**, **f**) (*P ≤ 0.05, ***P ≤ 0.001).

**Fig. 2:**
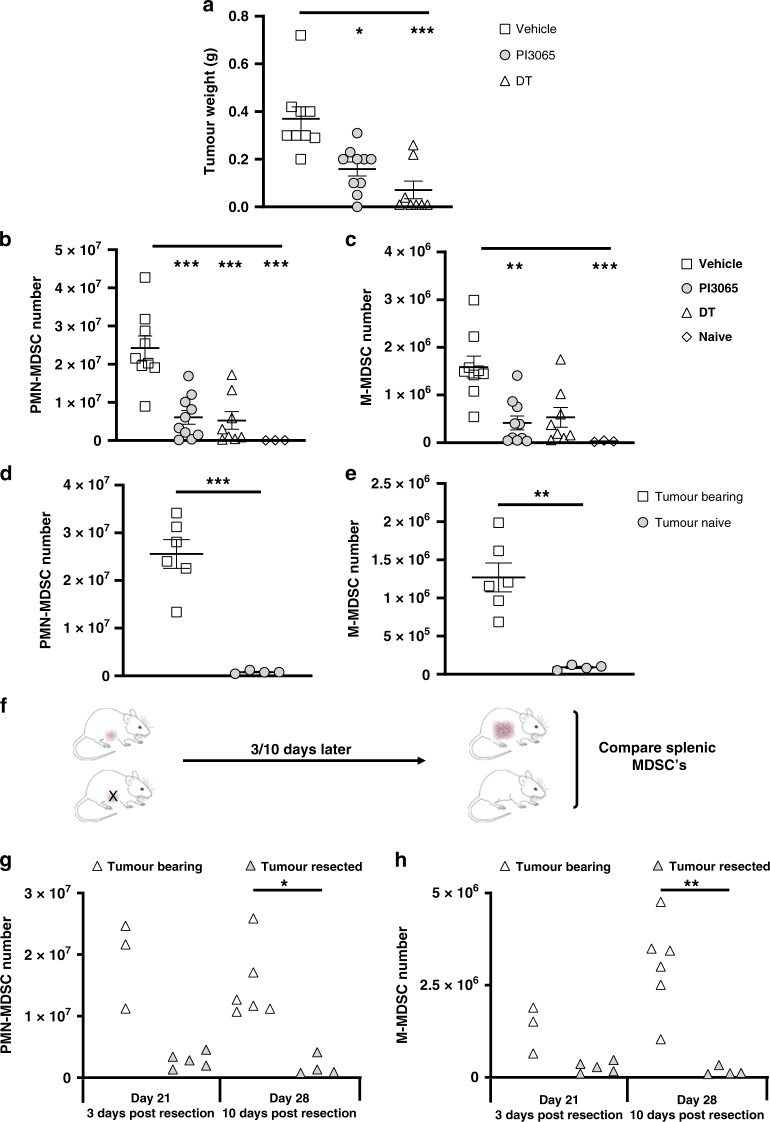
Tumours promote and support the expansion of splenic MDSC populations via Treg-independent mechanisms. **a** Tumour weight at day 21 post tumour injection from WT mice treated with either vehicle or PI-3065 or DEREG mice treated with DT (8–10 mice/group). The number of splenic (**b**) PMN-MDSCs and (**c**) M-MDSCs from WT mice treated with either vehicle or PI-3065 or DEREG mice treated with DT (3–10 mice/group). The total number of (**d**) PMN-MDSCs and (**e**) M-MDSCs in the spleens of tumour-bearing or tumour-naive nude mice at day 28 post tumour injection (4–6 mice/group). **f** Schematic illustrating the surgical resection time-course utilised. Total numbers of (**g**) PMN-MDSC and (**h**) M-MDSC in either tumour-bearing mice at day 21 and day 28 post tumour injection or at the equivalent day’s post tumour resection (3–6 mice/group). All data are displayed as the mean ± SEM. Statistical significance was determined by Kruskal–Wallis test (**a**–**c**, **g**, **h**) and by unpaired *t* test (**d**, **e**) (*P ≤ 0.05, ***P ≤ 0.001).

**Fig. 3:**
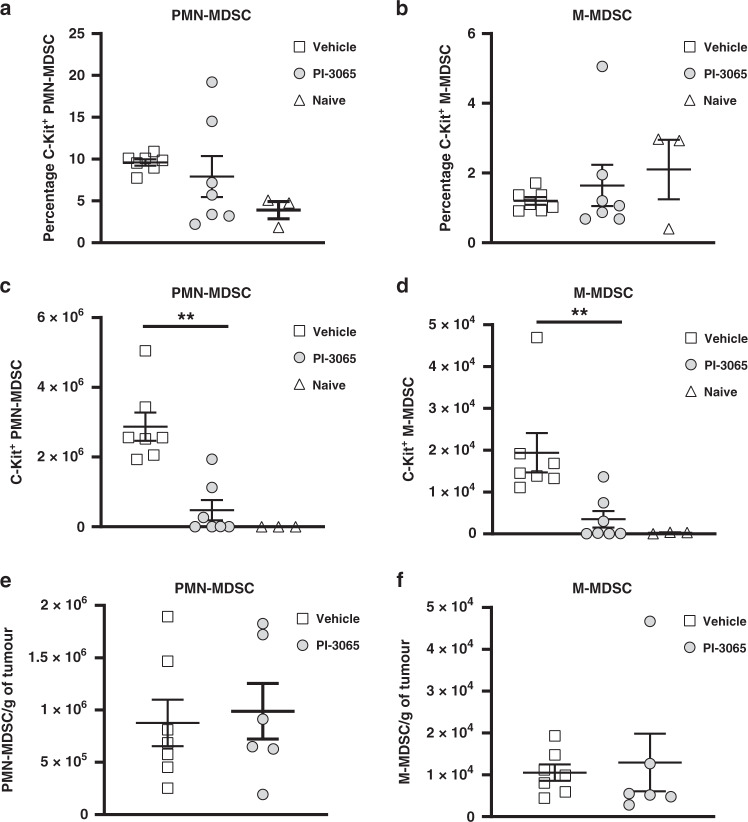
PI3Kδ inhibition does not reduce c-KIT expression or tumoural MDSC recruitment. The percentage of splenic (**a**) PMN-MDSC and (**b**) M-MDSC expressing c-KIT from vehicle or PI-3065 treated mice at day 28 post tumour injection (3–7 mice group). The total number of splenic (**c**) PMN-MDSCs and (**d**) M-MDSCs expressing c-KIT from vehicle- or PI-3065-treated mice at day 28 (3–7 mice/group). Total tumoural (**e**) PMN-MDSC and (**f**) M-MDSC infiltration normalised to gram of tumour weight at day 21 (6–7 mice/group). All data are displayed as the mean ± SEM. Statistical significance was determined by Kruskal–Wallis test (**a**–**d**) and unpaired *t* test (**e**, **f**) (**P ≤ 0.01).

**Fig. 4:**
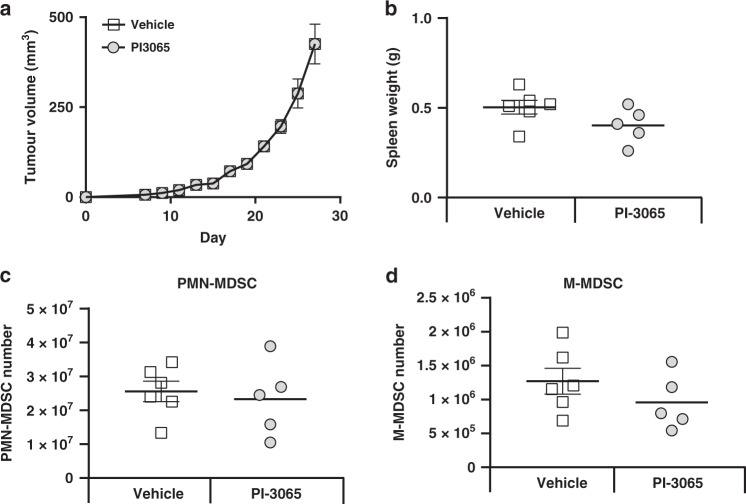
Successful tumour control driven by PI3Kδ inhibition is dependent on T cells and not MDSCs. **a** Tumour growth curves of nude mice treated with either vehicle or PI-3065 (5–6 mice group). **b** Spleen weight of nude mice treated with either vehicle or PI-3065 at day 28 post tumour injection (5–6 mice/group). The total number of splenic (**c**) PMN-MDSCs or (**d**) M-MDSCs from nude mice treated with either vehicle or PI-3065 at day 28 post tumour injection. Statistical significance was determined by unpaired *t* test.

**Fig. 5:**
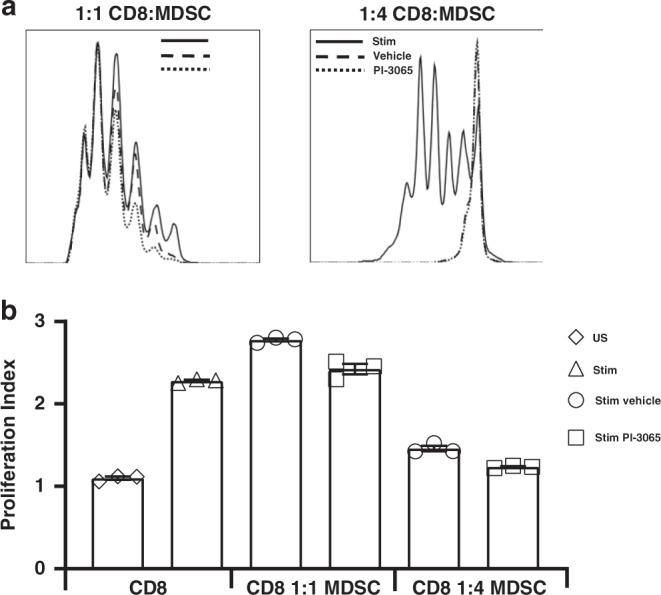
PI3Kδ inhibition does not reduce MDSC immunosuppression ex vivo. **a** Representative flow cytometry histogram of CD3^+^ CD28^+^ antibody stimulated CD8^+^ T cells cultured with MDSCs from vehicle or PI-3065-treated mice at a ratio of 1:1 or 1:4. **b** Proliferation index of CD8^+^ T cells when stimulated with anti-CD3 and CD28 antibodies alone or in the presence of MDSCs at either a ratio of 1:1 or 1:4 (CD8 T cell: MDSCs). Experiments were performed in triplicate and repeated twice.

In figures 1–4, the statistical differences between groups were assessed for normality to determine if a parametric or non-parametric statistical test should be employed. For normally distributed datasets, either an unpaired *t* test was used to compare two groups or for multiple groups, one-way ANOVA with a Tukey’s multiple comparison post hoc test was performed. For non-normally distributed datasets, a Mann–Whitney *t* test was used to compare between groups and a Kruskal–Wallis test with Dunn’s multiple comparison post hoc test used to compare between multiple groups. *P* values of ≤0.05 were considered significant, with values of ≤0.01 considered highly significant. The number of mice used and replicates is detailed for each figure in the corresponding figure legend. The proliferation index used in Fig. 5 was calculated using the following formula; (total number of cell divisions/cells that went into division).

## Results

### PI3Kδ inhibitor treatment leads to a significant reduction in splenic MDSCs

Upon subcutaneous inoculation of 4T1 breast cancer in mice expressing endogenously inactivated PI3Kδ, a significant reduction in spleen-associated expansion of MDSCs was observed [17]. Using the same 4T1 tumour model, we now sought to examine the impact on this MDSC expansion of a pharmacological approach to inhibiting PI3Kδ. Daily oral administration of the PI3Kδ-selective inhibitor PI-3065 [17] led to a significant control of tumour growth, accompanied by a significant reduction in the splenomegaly normally observed in mice bearing 4T1 tumours (Fig. 1a, b and Supplementary Fig. 1). Detailed flow cytometric analysis of the immune cells present in the spleens of tumour-bearing vehicle- or PI-3065-treated animals revealed that splenomegaly was driven by a 40-fold expansion of PMN-MDSCs (CD11b^+^ Ly6G^+^ Ly6C^lo^) in vehicle-treated tumour-bearing animals, and to a lesser extent by expansion of M-MDSCs (CD11b^+^ Ly6G^−^Ly6C^hi,^, 30-fold expansion) (Fig. 1c). NK cells also demonstrated a 14-fold expansion in both vehicle and PI-3065-treated animals, but when total numbers of cells were compared in the spleen, the dominant population driving splenomegaly was the PMN-MDSC population (Fig. 1d). PI-3065 treatment significantly reduced the splenic expansion of both MDSC subpopulations by day 28 post tumour injection (Fig. 1e, f). Although PI3Kδ inhibitors, such as Idelalisib have been used as a clinical therapy in chronic lymphocytic leukaemia (CLL), we saw no significant impact of PI-3065 on B-cell numbers in the spleen.

### MDSC expansion is driven by tumour growth

The reduction in splenic MDSCs was associated with reduced Treg numbers and reduced tumour volume in PI-3065-treated mice, whilst vehicle mice exhibited a significantly elevated frequency of splenic MDSCs, high frequencies of Treg and larger tumours. These findings are in line with our previous study, which showed that administration of PI-3065 results in Treg inactivation, unleashing an anti-tumour CD8^+^ T-cell response and altering the ratio of CD8:Tregs in favour of tumour control [17, 18].

With these data in mind, we hypothesised that the effect of the PI3Kδ inhibitor on MDSC was mediated indirectly via effects on Treg and/or tumour growth. To test this, we first examined MDSC numbers in mice where Treg were depleted by administration of diphtheria toxin (DT) to DEREG mice [21]. We found a comparable reduction in tumour size and fewer MDSCs in the spleen upon DT-induced Treg depletion in tumour-bearing DEREG mice as upon treatment with PI-3065 (Fig. 2a–c), supporting the hypothesis that MDSC expansion is profoundly influenced by the control of tumour growth following Treg depletion. To determine whether tumour or Treg was the major driver of MDSC expansion, we measured MDSC in tumour-bearing nude mice. These data clearly indicate that the tumour promotes MDSC expansion in the absence of Tregs (Fig. 2d, e).

To confirm the importance of the tumour in expanding and maintaining splenic MDSCs we employed a tumour resection model in vehicle-treated, Treg-replete mice. We injected mice with tumours at day 0, and then split the mice into two groups; mice undergoing surgical tumour resection and mice whose tumours would continue to grow (Fig. 2f). We observed that following tumour resection at day 21, the numbers of both PMN-MDSCs and M-MDSCs were dramatically reduced in the spleen at day 3 post excision compared to animals with a tumour in situ (Fig. 2g, h). In the case of tumours that continued to grow, the MDSC numbers also expanded, however, in mice whose tumours had been removed, the numbers of MDSCs in the periphery remained low. These data confirm that the presence of tumour is critical for the expansion and maintenance of peripheral MDSCs and are compatible with previous studies demonstrating that 4T1 tumours, through the secretion of stem cell factor (SCF) [22] promote PMN-MDSC expansion via c-kit receptor signalling [23, 24]. We found that whilst the proportion of c-Kit^+^ MDSCs was similar in small (treated) and large (control) tumours (Fig. 3a, b), there was a reduction in the absolute number of c-Kit^+^ PMN-MDSCs and M-MDSCs, corresponding with fewer overall MDSCs in the spleen (Figs. 1e, f and  3c, d). To assess the impact of reduced splenic MDSC numbers on tumoural MDSC recruitment we examined small (treated) and large (control) tumours at day 21 post tumour injection. Flow cytometric analysis revealed no difference in tumoural MDSC infiltration when normalised per gram of tissue (Fig. 3e, f), indicating that the impact of PI-3065 treatment upon MDSC is inhibition of splenic expansion rather than intra-tumoural recruitment.

### Inhibition of PI3Kδ does not directly affect MDSC numbers

Whilst the above data support the premise that tumours drive MDSC expansion and that inhibition of tumour growth via Treg depletion controls this expansion, they do not rule out direct effects of PI3Kδ-inhibition on MDSCs. Indeed, studies to date have suggested that MDSCs require signalling via PI3Kδ for maximal suppressive effect [17]. In addition, studies indicate that inhibition of PI3K using the pan-PI3K inhibitor LY294002 can reduce c-KIT expression on hematopoietic cells [25, 26] thereby indicating a possible mechanism whereby PI3Kδ inhibition could directly impinge on MDSC numbers.

To determine if targeting the PI3Kδ signalling pathway affected MDSC numbers in the absence of direct effects on Tregs or tumours, we injected tumours into BALB/C Nude mice and treated with either vehicle or PI-3065. PI-3065 treatment in the absence of T cells conferred no tumour control (Fig. 4a). Analysis of spleens at day 28, post injection, revealed comparable spleen weights (Fig. 4b) and PMN-MDSC and M-MDSC populations (Fig. 4c, d). Taken together, these data indicate that PI-3065 treatment does not directly target MDSCs in vivo.

### MDSC immunosuppression is not affected by PI3Kδ inhibition

We have previously demonstrated that the addition of PI-3065 to splenocytes has a direct effect on CD8^+^ T-cell proliferation in vitro [18], thus a different approach was needed to examine the suppressive capacity of MDSC exposed to PI3Kδ inhibition. To establish if therapeutic administration of PI-3065 resulted in the development of MDSCs with inferior suppressive capacity in tumour-bearing hosts, ex vivo suppression assays were performed. Splenic CD8^+^ T cells from naive mice were stimulated with anti-CD3 and CD28 antibodies and co-cultured with MDSCs isolated from tumour-bearing vehicle- or PI-3065-treated mice. At a ratio of 1:1, neither vehicle- or PI-3065-derived MDSCs had a notable effect on CD8^+^ T-cell proliferation (Fig. 5a, b). At a higher ratio of 4 MDSCs per T cell, we observed that both vehicle- and PI-3065-treated MDSCs reduced CD8^+^ T-cell proliferation. However, there was no significant difference between MDSCs derived from either vehicle- or PI-3065-treated mice, implying no direct effect of the inhibitor on MDSC activity.

## Discussion

Previous studies have demonstrated that both M-MDSCs and PMN-MDSCs are dependent on PI3K signalling for optimal immunosuppression [20]. Genetic inactivation of PI3Kδ in mice resulted in both reduced numbers of peripheral MDSCs in tumour-bearing hosts and ex vivo reduced capacity to suppress T-cell proliferation [17]. As an extension of these studies, we sought to determine whether pharmacological inhibition of PI3Kδ using the small molecule inhibitor PI-3065, also targets MDSCs therapeutically. We observed that animals treated with PI-3065 had reduced tumour burden and that in both vehicle- and drug-treated animals, tumour size correlated with splenomegaly driven by MDSC expansion. Following surgical removal of tumours in untreated mice, the numbers of MDSCs reduced dramatically within 3 days, to almost negligible levels, indicating that MDSC numbers are largely controlled by tumour burden and not by administration of PI-3065 per se. Studies in gastric cancer have recently demonstrated increased MDSC numbers in the spleen and peripheral blood that correlate with tumour stage [27, 28]. Furthermore, the expansion of MDSCs in the peripheral blood of breast cancer patients is reversed following surgical excision of the primary tumour [4], demonstrating that the tumour is critical for supporting MDSCs in the periphery.

Tregs and MDSCs are known to play reciprocal roles within the tumour microenvironment, with MDSCs promoting de novo induction of Treg [10, 11], whilst Tregs can subsequently promote MDSC expansion [29, 30]. Selective depletion of Tregs in tumour-bearing DEREG mice resulted in both reduced tumour burden and splenic MDSC expansion comparable with PI-3065 treatment. However, tumour-driven MDSC expansion was observed in nude mice which lack Treg indicating that it is the tumour and not Tregs that drives this expansion. Splenic MDSC expansion was comparable in PI-3065- and vehicle-treated nude mice indicating no direct effect of the PI3Kδ-inhibitor on MDSC. Overall, these data demonstrate that the tumour, and not Treg or inhibition of PI3Kδ signalling, is the most significant influence on splenic MDSC numbers.

Finally, given that Ali and colleagues previously demonstrated impaired immunosuppression by PI3Kδ genetically deficient MDSCs [17], we sought to address if MDSC suppression in vivo could be ameliorated by pharmacological PI3Kδ blockade. Using MDSCs isolated from PI-3065-treated mice, we observed equivalent immunosuppression on a per cell basis ex vivo. Although a caveat of this approach is that the in vivo effects of PI-3065 on MDSC may be reversed in ex vivo cultures, meaning that we cannot conclude that the MDSCs present in PI-3065-treated mice are unaffected functionally, the data does indicate that inhibition of PI3Kδ in vivo does not result in an irreversible block in the suppressor function of these cells. This finding is compatible with data published by Davis and colleagues, who demonstrated that a reduction in MDSC suppression could only be achieved with a dual PI3Kδ/γ inhibitor, IPI-145 [31]. As MDSCs are known to employ both the PI3Kδ and γ isoforms for downstream signalling (reviewed in ref. [14]), it is feasible that pharmacological targeting of PI3Kδ alone, is not sufficient to inhibit their suppressive mechanisms. Gyori et al. and colleagues reported similar findings in the MC38 colon carcinoma model, whereby T-cell-mediated control of tumour growth could only be achieved if treatment with the PI3Kδ inhibitor, Idelalisib, which resulted in reduced intra-tumoural Treg numbers, was combined with a CSF1R inhibitor to reduce numbers of tumour associated macrophages [32].

The tumour itself is central to the immunosuppression mediated by MDSCs, as removal of the tumour dramatically reduces the peripheral MDSC population, enabling the establishment of a feedback loop favouring tumour rejection. Alleviation of immunosuppression by surgical removal of tumours has also been reported in patients with colorectal cancer [33], highlighting the critical role the tumour plays in amplifying immunosuppressive pathways. Thus, whilst inactivation of Treg appears to be the primary effect of PI-3065 treatment, our data indicate that through initiating immune-mediated control of tumour growth, a cascade of events is unleashed which serve to break down immunosuppressive pathways, further enhancing tumour immunity.

## Supplementary information

aj checklist

Supplementary Figure 1

## Data Availability

All data generated or analysed during this study are included in this published article.
