# Supplementary material for: Treg-driven tumour control by PI3Kδ inhibition limits myeloid-derived suppressor cell expansion
Source: Br J Cancer. 2022 Aug 19;127(9):1595–602. doi: 10.1038/s41416-022-01917-0 (PMC9596434; doi:10.1038/s41416-022-01917-0)
Supplement: Supplementary file 2 — Supplementary Figure 1 [file 41416_2022_1917_MOESM2_ESM.pdf]

Supplementary Figure 1

A **B**

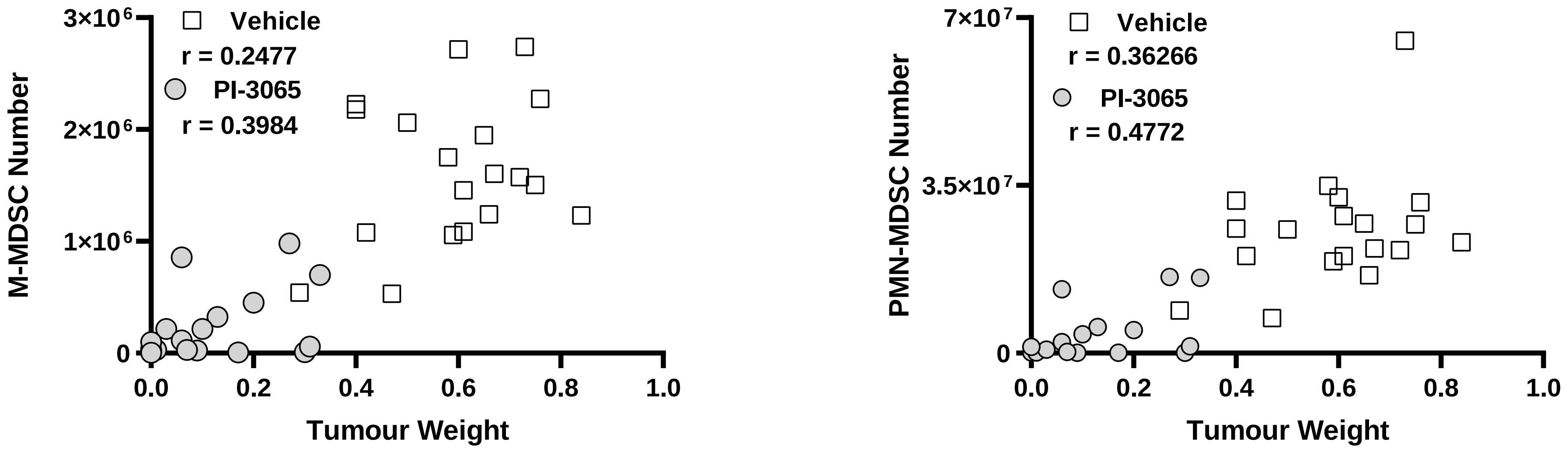

Splenic MDSC Expansion is driven by increased tumour size
Total numbers of (A) M-MDSC and (B) PMN-MDSC compared with tumour weight at day 28 post-tumour injection (18-19 mice/group). Statistical significance was determined by linear regression and r2 values provided.
